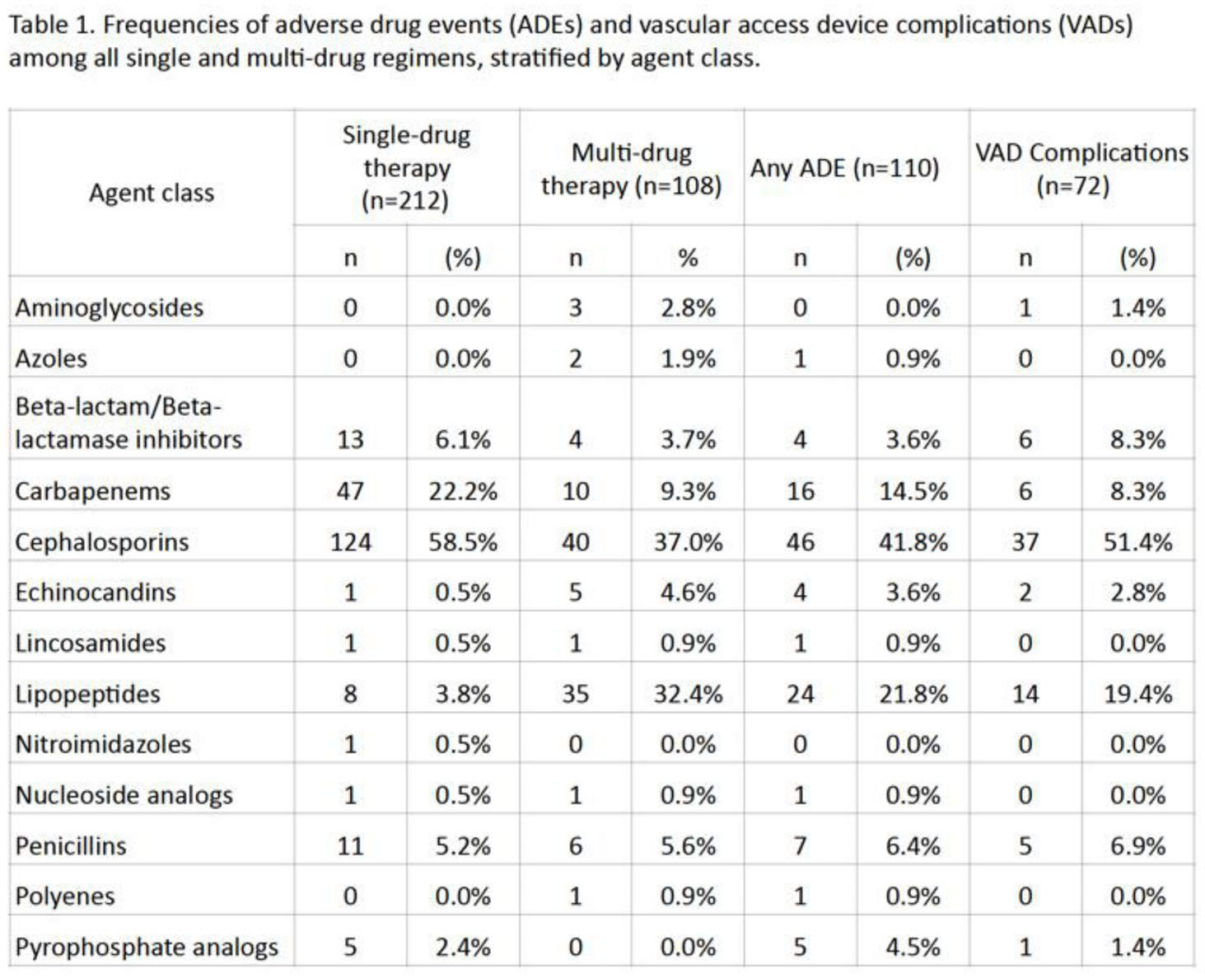# Retrospective cohort analysis of the safety of outpatient parenteral antimicrobial therapy (OPAT) in an academic hospital

**DOI:** 10.1017/ash.2022.170

**Published:** 2022-05-16

**Authors:** Kaylyn Billmeyer, Alison Galdys, Susan Kline, Elizabeth Hirsch, Jennifer Ross, Michael Evans

## Abstract

**Background:** Although many infectious conditions can be safely treated with oral antimicrobials, select circumstances require parenteral antimicrobial therapy. Benefits of OPAT include prevention of hospital-associated conditions and significant cost savings. However, risks of OPAT include adverse drug events (ADEs) and vascular access device (VAD) complications. We analyzed the safety of OPAT regimens as part of implementing a collaborative OPAT program. **Methods:** We reviewed adult patients discharged home from an academic hospital between January 2019 and June 2021. Patients with cystic fibrosis were excluded. Data on OPAT agents, ADEs, and VAD complications were collected from electronic medical records by 2 reviewers using a standardized REDCap instrument. The institutional review board approved this study. **Results:** The cohort comprised 265 unique patients; 212 (80%) received single-drug therapy and 53 (20%) received multidrug therapy. In total, 81 patients (31%), who received a total of 110 antimicrobials, experienced an ADE. In total, 55 patients (21%), who received a total of 72 antimicrobials, experienced a VAD complication. Patients who received >1 antimicrobial were more likely to experience an ADE (53% vs 25%; *P* = .0002) or a VAD complication (32% vs 18%; *P* = .04). Cephalosporins were the most frequently prescribed antimicrobial class (Table 1). **Conclusions:** ADEs and VAD complications were frequent in patients on OPAT. Local data should inform (1) the selection of OPAT therapy and (2) the standardized monitoring of patients who receive OPAT going forward in the implementation of this collaborative OPAT program.

**Funding:** None

**Disclosures:** None

**Table:**